# A new fluorescent probe for colorimetric and ratiometric detection of sulfur dioxide derivatives in liver cancer cells

**DOI:** 10.1038/srep45294

**Published:** 2017-03-28

**Authors:** Dong-Peng Li, Zhao-Yang Wang, Jie Cui, Xin Wang, Jun-Ying Miao, Bao-Xiang Zhao

**Affiliations:** 1 Institute of Organic Chemistry, School of Chemistry and Chemical Engineering, Shandong University, Jinan 250100, P.R. China; 2 Institute of Developmental Biology, School of Life Science, Shandong University, Jinan 250100, P.R. China

## Abstract

A new ratiometric fluorescent probe was constructed with hemicyanine and 7-nitrobenzofurazan for detection of sulfur dioxide derivatives (HSO_3_^−^/SO_3_^2−^). The ratiometric response mode could be attributed to the efficient FRET (Förster resonance energy transfer) platform. The probe exbihited some desirable properties including fast response (within 2 minutes), good selectivity and high sensitivity. Moreover, the probe could detect endogenous HSO_3_^−^ in liver cancer cells rather than normal liver cells, implying the diagnosal potential of the probe.

Sulfur dioxide is one of the major origins of acid rain, putting serious impacts on water and soil^1^. People who ever exposed to high levels of sulfur dioxide might suffer respiratory and cardiovascular diseases, lung cancer or neurological disorders^2^. However, research revealed that sulfur dioxide could be produced in cytosols and mitochondria of cells^3^. The main raw materials for sulfur dioxide generation in cells are hydrogen sulfide (H_2_S) and sulfur-containing amino acids^3^. On account of its functions in many physiological processes and physiopathology, sulfur dioxide was recognized as a new gaseous transmitter^4^. However, study of sulfur dioxide in living systems is still in its infancy. Lacking reliable analytical methods is one of the main bottlenecks.

Sulfur dioxide can easily dissolve in water (94 mg mL^−1^, 25 °C) to form sulfite (SO_3_^2−^) and bisulfite (HSO_3_^−^) anions. So the physiological effects of sulfur dioxide can be attributed to its derivatives (HSO_3_^−^/SO_3_^2−^). Traditionally, many methods such as titrimetry, chromatography, electrochemistry and capillary electrophoresis are available for the detection of HSO_3_^−^/SO_3_^2− ^5,6,7,8. However, these methods usually suffered long operation time, low sensitivity, tedious operation and no applications inside living cells. Alternatively, fluorometric analysis has emerged in recent years, which could be used to detect analytes with high sensitivity and high accuracy^9^,^10^. More importantly, *in situ* and real-time imaging of analytes could be carried out by non-destructive fluorescent probes^11^,^12^.

Among fluorescent probes, ratiometric ones are more desirable than single intensity-based ones due to built-in correction of the two emission bands^13^,^14^. By now, two detection mechanisms have been reported in the design of fluorescent probes for HSO_3_^−^/SO_3_^2−^: reactions with HSO_3_^−^/SO_3_^2−^ and hydrogen-bond formation with HSO_3_^−^/SO_3_^2^,^3^,^4^,^5^,^6^,^7^,^8^,^9^,^10^,^11^,^12^,^13^,^14^,^15^,^16^. The reactions with HSO_3_^−^/SO_3_^2−^ include cleavage of levulinate group^17^,^18^, nucleophilic addition to aldehyde group^19^,^20^ or “C=C” double bond^21^,^22^,^23^,^24^,^25^,^26^,^27^,^28^,^29^,^30^,^31^. However, the probes based on hydrogen-bond formation were sensitive to environment; The probes based on cleavage of levulinate group took too long (usually 20–60 minutes) to detect HSO_3_^−^/SO_3_^2−^ in real time; The probes based on nucleophilic addition to aldehyde group could only function well under acidic conditions (usually pH = 5). So we concentrated on developing new favorable probes by using nucleophilic addition reaction with “C=C” double bond.

Here a new ratiometric fluorescent probe (HCy-NBD) was constructed by connecting hemicyanine and 7-nitrobenzofurazan with a piperazine moiety as the non-conjugate “bridge”. Both the good overlap of the two bands (fluorescence emission band of 7-nitrobenzofurazan fluorophore and absorption band of hemicyanine fluorophore) and the proper space distance between the two fluorophores benefit the FRET process in HCy-NBD. As a result, the 7-nitrobenzofurazan fluorophore may emit very weak fluorescence while the hemicyanine fluorophore may emit strong fluorescence. Upon nucleophilic addition of HSO_3_^−^/SO_3_^2−^ to the hemicyanine fluorophore, the conjugated system was broken and the FRET process was blocked, restoring the fluorescence of the 7-nitrobenzofurazan fluorophore. The proposed sensing mechanism was shown in Fig. 1.

## Results and Discussion

### Response of probe HCy-NBD toward bisulfite in aqueous solution

**Figure 2:**
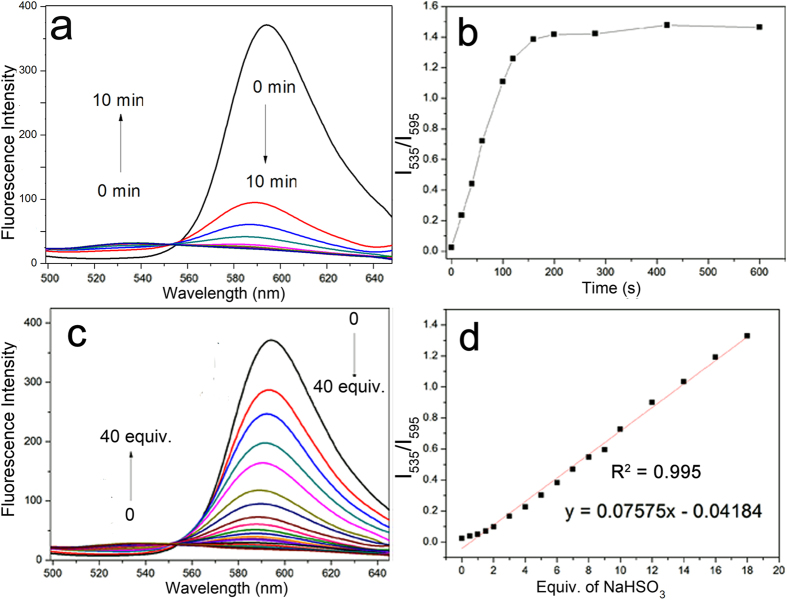
Fluorescence response of probe HCy-NBD toward NaHSO_3_. (**a**) and (**b**) Fluorescence changes of HCy-NBD in the presence of NaHSO_3_ (25 equiv.) in 10 min. (**c**) Fluorescence titration spectra of HCy-NBD upon addition of NaHSO_3_ (0–40 equiv). (**d**) The linear relationship between I_535_/I_595_ and NaHSO_3_ (0–18 equiv.). [HCy-NBD] = 10 μM; Buffer: 50 mM Tris-HCl containing 40% ethanol; λex = 345 nm, slit: 10 nm/12 nm.

NaHSO_3_ was used as donor of HSO_3_^−^/SO_3_^2−^. In the presence of 25 equiv. of NaHSO_3_, the emission band peaked at 595 nm decreased gradually, while a new band peaked at 535 nm gradually increased. The intensity ratios of the two emission bands (I_535_/I_595_) changed by a 61-fold from 0.024 to 1.47. The space between the two bands was much wider to avoid the overlap (Supplementary Table 1)^32^. The reaction could complete rapidly (in 2 min), which was suitable for real-time detection (Fig. 2, Supplementary Fig. 1). Upon addition of NaHSO_3_ (0–40 equiv.) to the buffer solution of the probe, I_535_/I_595_ changed quantitatively depending on NaHSO_3_ concentrations (Fig. 2, Supplementary Fig. 2). A good linear relationship between I_535_/I_595_ and the concentrations of NaHSO_3_ (0–18 equiv.) was observed. Based on the linearity, the detection limit was determined to be 68 nM (S/N = 3), which was superior to many reported probes^33^,^34^,^35^.

**Figure 3:**
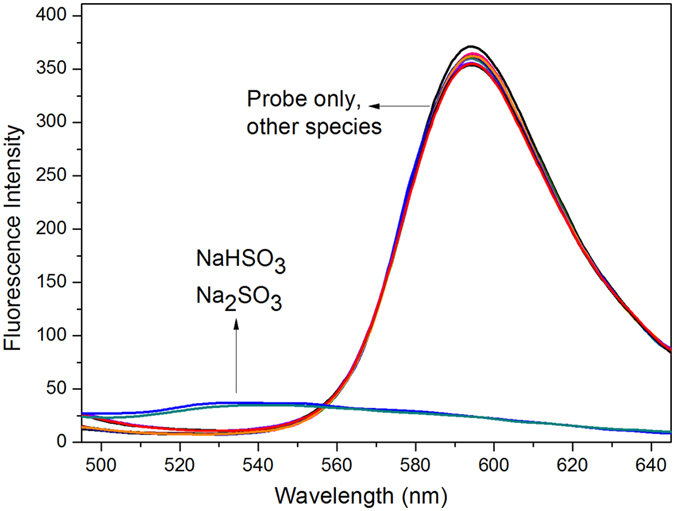
Fluorescence response of probe HCy-NBD toward various species. Species containing F^−^, Cl^−^, Br^−^, I^−^, HCO_3_^−^, NO_3_^−^, SO_4_^2−^, ClO^−^, H_2_O_2_, CN^−^, SCN^−^, S_2_O_3_^2−^, HS^−^, Cys, Hcy, GSH, HSO_3_^−^ and SO_3_^2−^ were involved. Final concentration for all the species was 250 μM except for Cys, Hcy and GSH (1 mM). λex = 345 nm, slit: 10 nm/12 nm.

Among various anions and biothiols, only NaHSO_3_ and Na2SO_3_ could lead to naked-eye changes in the colour of probe solutions (Supplementary Fig. 3). This implied the potential of probe HCy-NBD for colorimetric and selective detection of HSO_3_^−^/SO_3_^2−^. The good selectivity was further verified by fluorescence measurements (Fig. 3, Supplementary Fig. 4). Common biological relevant anions and small molecules including CN^−^, HS^−^ and biothiols hardly brought about significant fluorescence changes. The good selectivity could be well understood by the strong nucleophilic ability of HSO_3_^−^/SO_3_^2−^ under neutral conditions. It was reported that HS^−^ could react with 7-nitrobenzofurazan moiety to quench fluorescence^36^. So compound Donor (Supplementary Scheme 1) was used to reveal the reactivity toward HS^−^. Results showed that the fluorescence of compound Donor was almost unaffected, even with 100 equiv. HS^−^ for up to 1 h (Supplementary Fig. 5). The reactivity of probe HCy-NBD toward NaHSO_3_ was almost unaffected in the presence of various species (Supplementary Fig. 6).

### Mechanisms

The solution pH could usually put large impacts on the detection by affecting the state of reactants (NaHSO_3_ and HCy-NBD) or the reaction efficiency between them^37^. The fluorescence of probe HCy-NBD was almost constant in the range of pH 4–8. The stronger the basicity of the solution, the easier the reaction between probe HCy-NBD and NaHSO_3_. (Supplementary Fig. 7). So it was the nucleophilicity of NaHSO_3_ that was mainly affected by changing its existence forms^38^.

**Figure 4:**
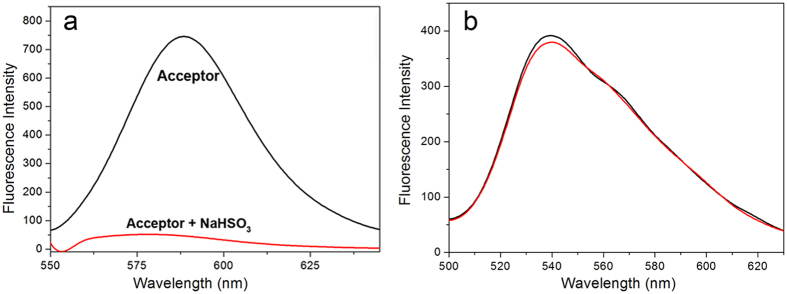
Fluorescence spectra of compound Acceptor and compound Donor in the presence of NaHSO_3_. Fluorescence of compound Acceptor (**a**) and compound Donor (**b**) in the absence or presence of NaHSO_3_. NaHSO_3_ was 20 equiv. to that of Donor or Acceptor. λex = 345 nm for the Donor and λex = 530 nm for the Acceptor, slit: 10 nm/12 nm.

**Figure 5:**
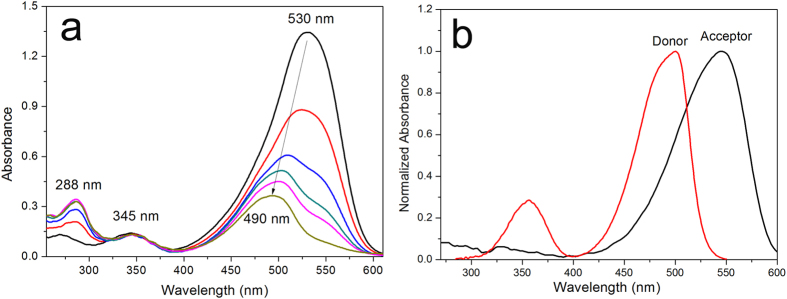
UV-vis spectra of HCy-NBD. (**a**) Absorption spectra of HCy-NBD (10 μM) in the presence of different amounts of NaHSO_3_ (0–300 μM). (**b**) Normalized absorption spectra of compound Donor and Acceptor.

Hemicyanine moiety is electrophilic and is liable to react with nucleophilic HSO_3_^−^/SO_3_^2−^. Upon addition of NaHSO_3_, the fluorescence emission band of the Acceptor (Supplementary Scheme 1) diminished but that of the Donor was unaffected (Fig. 4). Furthermore, the UV-vis absorption spectra of probe HCy-NBD in the presence of NaHSO_3_ (0–300 μM) were recorded (Fig. 5). The absorption band peaked at 530 nm which was assigned to hemicyanine moiety decreased and shifted to 490 nm which should belong to the absorption band of the 7-nitrobenzofurazan moiety (Fig. 5b). Meanwhile, a new band centred at 288 nm appeared. High-resolution mass spectroscopy revealed two dominant peaks at m/z 509.2304 and 591.2103, which could be ascribed to [HCy-NBD + H]^+^ and [HCy-NBD + HSO_3_^−^]^+^, respectively. (Supplementary Fig. 8). ^1^H NMR titration experiment also revealed the proposed reaction mechanism. The two peaks at about 8.3 and 7.3 ppm, which could be attributed to protons on the C=C double bond of the probe, shifted to 4.8 and 4.9 ppm after reaction with Na_2_SO_3_ (Supplementary Scheme 2). Thus HSO_3_^−^/SO_3_^2−^ was captured by the hemicyanine moiety via Michael addition reaction, which was in consistence with literatures^27^,^39^.

**Figure 6:**
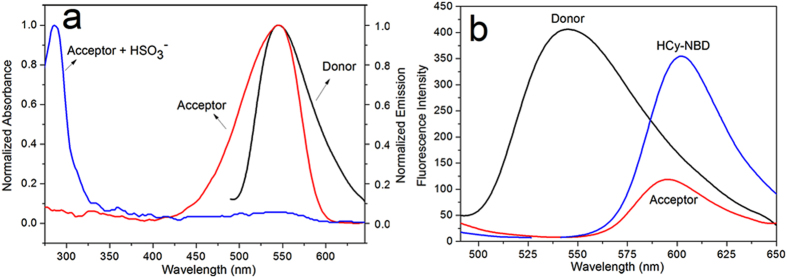
Interpretation of FRET process in HCy-NBD. (**a**) Normalized fluorescence spectrum of the Donor; Normalized UV-vis absorption spectrum of the Acceptor before and after addition of NaHSO_3_ (20 equiv.). (**b**) Fluorescence spectra of the Donor, Acceptor and probe HCy-NBD. λex = 345 nm, slit: 10 nm/12 nm. [Donor] = 5 μM, [Acceptor] = [HCy-NBD] = 10 μM.

Absorption spectra of compound Acceptor overlapped greatly with the fluorescence spectra of compound Donor (Fig. 6a). Thus the fluorescence of the 7-nitrobenzofurazan moiety would be quenched because of the energy transfer to the hemicyanine moiety via FRET process^40^,^41^. The FRET efficiency was determined to be 0.72. Interruption of the conjugated system in the hemicyanine moiety led to its absorption band blue-shifted greatly. So the abovementioned essential spectral overlap disappeared, and 7-nitrobenzofurazan moiety would emit fluorescence as a result. From the view of fluorescence intensity, the fluorescence of probe HCy-NBD was much greater than that of compound Acceptor excited at 345 nm, which further confirmed the FRET process in HCy-NBD (Fig. 6b).

### Applications of probe HCy-NBD in living cells

**Figure 7:**
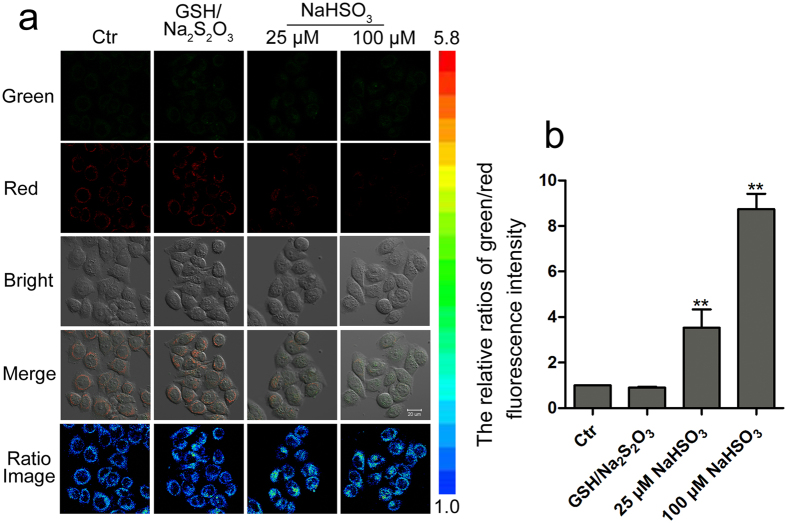
Detection of bisulfite in living L-02 cells. (**a**) Fluorescence, bright field and ratio images of L-02 cells which were incubated with HCy-NBD (5 μM) for 1 h, then with GSH (500 μM)/Na_2_S_2_O_3_ (500 μM) or NaHSO_3_ (25, 100 μM) for another 0.5 h. (**b**) The relative ratios of green/red fluorescence intensity. Images were acquired from 405–555 nm for green fluorescence, and from 560–700 nm for red fluorescence.

Probe HCy-NBD showed excellent photostability in living cells (Supplementary Fig. 9) and ignorable cytotoxicity (Supplementary Fig. 10). Thus probe HCy-NBD could be suitable for HSO_3_^−^/SO_3_^2−^ detection in living cells with minimum interference. Exogenous HSO_3_^−^ imaging experiments were primarily conducted in Hela cells. The ratios of fluorescence intensity (I_green_/I_red_) increased with the gradual increase of exogenous HSO_3_^−^ (Supplementary Fig. 11). L-02 cells treated with NaHSO_3_ (100 μM) and HCy-NBD (5 μM) showed 8-fold enhancement in the fluorescence intensity ratio compared with control group, which was much more sensitive than the previous reported probe HCy-D (Fig. 7)^32^.

**Figure 8:**
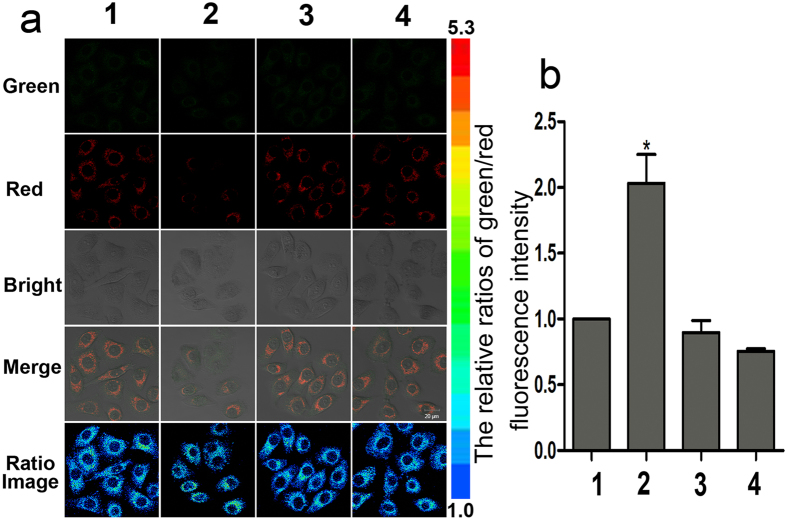
Detection of bisulfite in living HepG2 cells. (**a**) Row 1: HepG2 cells were incubated with HCy-NBD for 1 h; Row 2: HepG2 cells were incubated with HCy-NBD for 1 h, and then with GSH (500 μM) and Na_2_S_2_O_3_ (250 μM) for 0.5 h; Row 3: HepG2 cells were incubated with HCy-NBD for 1 h, then with TNBS (10 mM) for 0.5 h, GSH (500 μM) and Na_2_S_2_O_3_ (250 μM) for another 0.5 h; Row 4: HepG2 cells were incubated with HCy-NBD for 1 h, then with GSH (500 μM) for another 0.5 h. (**b**) The relative ratios of green/red fluorescence intensity of row 1, 2, 3 and 4 in (**a**). The ratio images were all obtained as F_green_/F_red_. Images were acquired from 405–555 nm for green fluorescence, and from 560–700 nm for red fluorescence, respectively. [HCy-NBD] 5 μM. λ_ex_ = 405 nm.

Endogenous HSO_3_^−^ could be produced with the help of TST enzyme^42^, which is widespread in nature and especially abundant in human liver cells^43^. HepG2 cells and L-02 cells were separately incubated with HCy-NBD (5 μM) for 1 h, followed by incubation with GSH (500 μM) and Na_2_S_2_O_3_ (250 μM) for another 0.5 h. Remarkable fluorescence change was observed only in HepG2 cells (Figs 7 and 8). By contrast, no significant flu rescence change was observed in HepG2 cells incubated with HCy-NBD and GSH. Moreover, HepG2 cells pre-treated with HCy-NBD (5 μM) and TNBS (10 mM, 2,4,6-trinitrobenzenesulphonate, known as a TST inhibitor) then with GSH (500 μM) and Na_2_S_2_O_3_ (250 μM) showed no significant fluorescence change (Fig. 8). These results indicated that the endogenous HSO_3_^−^ produced enzymatically in HepG2 cells was responsible for the fluorescence change.

On the whole, endogenous HSO_3_^−^ in liver cancer cells rather than in normal liver cells could be detected, which revealed a new diagnostic feature of liver cancer cells. Therefore, the new way based on cellular level could be promising in liver cancer diagnosis and pathogenesis study of liver cancer.

## Conclusions

A new fluorescent probe based on an FRET platform was reported. The probe could detect HSO_3_^−^/SO_3_^2−^ rapidly, sensitively and selectively. The probe also showed high energy transfer efficiency, good biocompatibility and high reactivity toward HSO_3_^−^/SO_3_^2–^. Endogenous bisulfite was successfully detected in liver cancer cells by the probe, which might pave a new way for liver cancer diagnosis and pathogenesis study of liver cancer.

## Methods

### Apparatus and chemicals

^1^ H NMR (300 or 400 MHz) and ^13^C NMR (100 MHz) spectra were recorded on a Bruker Avance 300 or 400 spectrometer using CDCl_3_, DMSO-*d*_6_ or D_2_O as solvent and tetramethylsilane (TMS) as an internal standard. HR-MS spectra were recorded on a Q-TOF6510 spectrograph (Agilent). IR spectra were recorded by use of the IR spectrophotometer VERTEX 70 FT-IR (Bruker Optics). Melting points were measured on an XD-4 digital micro-melting point apparatus. Thin-layer chromatography (TLC) was conducted on silica gel 60F_254_ plates (Merck KGaA) and column chromatography was conducted over silica gel (mesh 200–300). Fluorescence measurements were conducted on a Perkin-Elmer LS-55 luminescence spectrophotometer, and UV-vis spectra were recorded on a U-4100 UV-Vis-NIR Spectrometer (Hitachi). Quartz cuvettes with a 1 cm path length and 3-mL volume were involved in fluorescence and UV-vis absorption measurements. The pH values were measured by use of a PHS-3C digital pH-meter (YouKe, Shanghai). All reagents were purchased from J&K, Aladdin and Sinopharm Chemical Reagent Co. and used without further purification.

### Preparation for UV-vis absorption and fluorescence spectral measurements

Tris-HCl buffer (50 mM, pH 7.4) was used throughout. HCy-NBD was dissolved in DMSO to get the stock solution (1 mM). Distilled water was used to prepare stock solutions (1 mM) of NaF, NaCl, NaBr, KI, NaHCO_3_, KNO_3_, NaClO, Na_2_SO_4_, KSCN, Na_2_S_2_O_3_, NaHS, Na_2_SO_3_, NaHSO_3_,(n-Bu)_4_CN, cysteine, homocysteine and glutathione. Stock solutions of NaHSO_3_ and Na_2_SO_3_ were freshly prepared each time before use. Test solutions were prepared by placing the stock solution of HCy-NBD (100 μL) and an appropriate aliquot of each testing species solution into a 10-mL volumetric flask, and the solution was diluted to 10 mL with Tris-HCl buffer (50 mM, pH 7.4) containing 40% ethanol (v/v).

### Cell imaging

L-02 cells or HepG2 cells were cultured in a 6-well plate in Dulbecco’s modified Eagle’s medium (DMEM) supplemented with 10% fetal bovine serum in an atmosphere of 5% CO_2_ and 95% air at 37 °C. HCy-NBD was dissolved in DMSO to get the stock solution (10 mM) and diluted to 5 μM each time before use. L-02 cells or HepG2 cells were incubated with HCy-NBD (5 μM) for 1 h, then treated with exogenous substances. Subsequently, the cell images were taken under a confocal microscope (LSM 700) at emission channels of 405–555 nm (green channel) and 560–700 nm (red channel), respectively.

### Synthesis and characterization of probe HCy-NBD

To ethanol (10 mL) was added compound 3 (320 mg, 0.67 mmol) and compound 4 (140 mg, 0.70 mmol) at room temperature (Supplementary Scheme 1). Then the mixture was stirred at room temperature for 5 h. The solvent was removed under reduced pressure, then the residue was subjected to column chromatography on silica gel (CH_2_Cl_2_ : MeOH = 10:1 to 1:1) to afford a dark red powder (390 mg, 91.0%). mp: >300 °C. ^1^H NMR (DMSO-*d*_6_, 400 MHz) δ (ppm): 8.55 (d, J = 9.2 Hz, 1 H), 8.34 (d, J = 16 Hz, 1H), 8.14 (d, J = 8.8 Hz, 2H), 7.81–7.74 (m, 2H), 7.59–7.50 (m, 2H), 7.36 (d, J = 16 Hz, 1H), 7.08 (d, J = 8.8 Hz, 2H), 6.62 (d, J = 9.2 Hz, 1H), 4.39 (br, 4H), 4.02 (s, 3H), 3.96–3.93 (m, 4H), 1.77 (s, 6H); ^13^C NMR (DMSO-*d*_6_, 100 MHz) δ (ppm): 180.69, 154.30, 153.88, 145.95, 145.30, 143.26, 142.49, 136.79, 134.26, 129.22, 128.43, 124.00, 123.12, 121.58, 114.40, 13.26, 107.04, 103.27, 93,34, 51.63, 48.62, 44.98, 33.84; IR (KBr) cm^−1^: 3082, 3037, 2970, 2923, 2856, 1574, 1525, 1479, 1373, 1291, 1189, 1171, 1112, 1015, 995, 929. HRMS: m/z calculated for C_29_H_29_N_6_O_3_^+^: 509.2301, found: 509.2300.

## Additional Information

**How to cite this article:** Li, D.-P. *et al*. A new fluorescent probe for colorimetric and ratiometric detection of sulfur dioxide derivatives in liver cancer cells. *Sci. Rep.* **7**, 45294; doi: 10.1038/srep45294 (2017).

**Publisher's note:** Springer Nature remains neutral with regard to jurisdictional claims in published maps and institutional affiliations.

## Supplementary Material

Supplementary Information

## Figures and Tables

**Figure 1:**
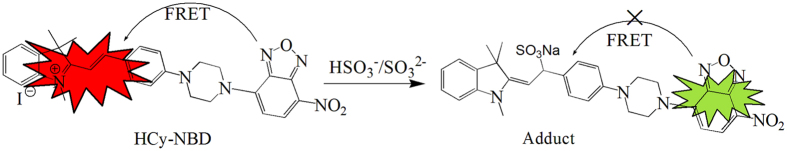
The structure of probe HCy-NBD and the proposed sensing mechanism.
