# Supplementary material for: A new fluorescent probe for colorimetric and ratiometric detection of sulfur dioxide derivatives in liver cancer cells
Source: Sci Rep. 2017 Mar 28;7:45294. doi: 10.1038/srep45294 (PMC5368602; doi:10.1038/srep45294)
Supplement: Supplementary Information [file srep45294-s1.doc]

**A new fluorescent probe for colorimetric and ratiometric detection of sulfur dioxide derivatives in liver cancer cells**

Dong-Peng Li^a^,, Zhao-Yang Wang^b^,, Jie Cui^a^, Xin Wang^a^, Jun-Ying Miao^b^, Bao-Xiang Zhao^a*^

*^a^ Institute of Organic Chemistry, School of Chemistry and Chemical Engineering, Shandong University, Jinan 250100, P.R. China. E-mail:* [*bxzhao@sdu.edu.cn*](mailto:bxzhao@sdu.edu.cn) *(B.X. Zhao); Tel.: +86 531 88366425; fax: +86 531 88564464;*

*^b^ Institute of Developmental Biology, School of Life Science, Shandong University, Jinan 250100, P.R. China.)*

**Calculation of energy transfer efficiency**

Energy transfer efficiency (*E*) was calculated using the following equation:

E = 1 - F_DA_ /F_D_

Where, *F_DA_* denotes the donor fluorescence with an acceptor, and *F_D_* denotes the donor fluorescence without an acceptor.

Cytotoxicity Assay

Hela cells were cultured in DMEM supplemented with 10% FBS in an atmosphere of 5% CO_2_ and 95% air at 37℃. The cells were placed in a 96-well plate, followed by addition of HCy-NBD with final concentrations of 1, 5 and 10 µM, respectively. The cells were then incubated for 6 h, followed by SRB assays.


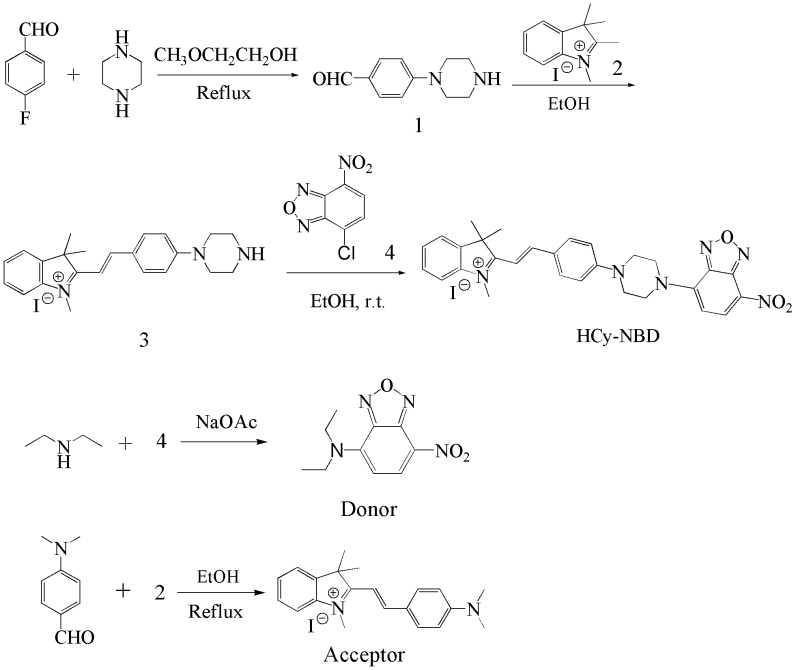


**Supplementary Scheme 1.** Synthesis of HCy-NBD, Donor and Acceptor.

**Compound 1, compound 3 and the Acceptor** was synthesized according to literature.^1^

**Synthesis of the Donor** To a mixture of NHEt_2_ (76 mg, 1 mmol) in dry CH_2_Cl_2_ (5 mL) was added NaOAc (21 mg), then compound 4 (211 mg, 1 mmol) dissolved in dry CH_2_Cl_2_ (10 mL) and absolute ethanol (6 mL) was added dropwise over a period of 30 min at 0℃. The mixture was stirred at room temperature for 5 h, after which the mixture was added into water (20 mL). The organic layer was separated, dried over anhydrous sodium sulfate and concentrated under reduced pressure. The residue was subjected to column chromatography on silica gel (petroleum ether : ethyl acetate = 8:1 to 4:1) to afford an orange solid (235 mg, 94%). Mp: 139-141℃. IR (KBr) cm^−1^: 3124, 2993, 2941, 2858, 1606, 1565, 1483, 1451, 1276, 1184, 1100, 1071, 996, 843, 804. ^1^H NMR (CDCl_3_, 300 MHz) δ (ppm): 8.44 (d, 1H, J = 9.3 Hz, ArH), 6.13 (d, 1H, J = 9.0 Hz, ArH), 3.98 (q, 4H, J = 6.6 Hz, N*CH_2_*CH_3_), 1.40 (t, 6H, J = 7.2 Hz, NCH_2_*CH_3_*); ^13^C NMR (CDCl_3_, 75 MHz) δ (ppm): 145.01, 144.68, 144.31, 135.54, 121.57, 100.72, 48.16, 12.44.


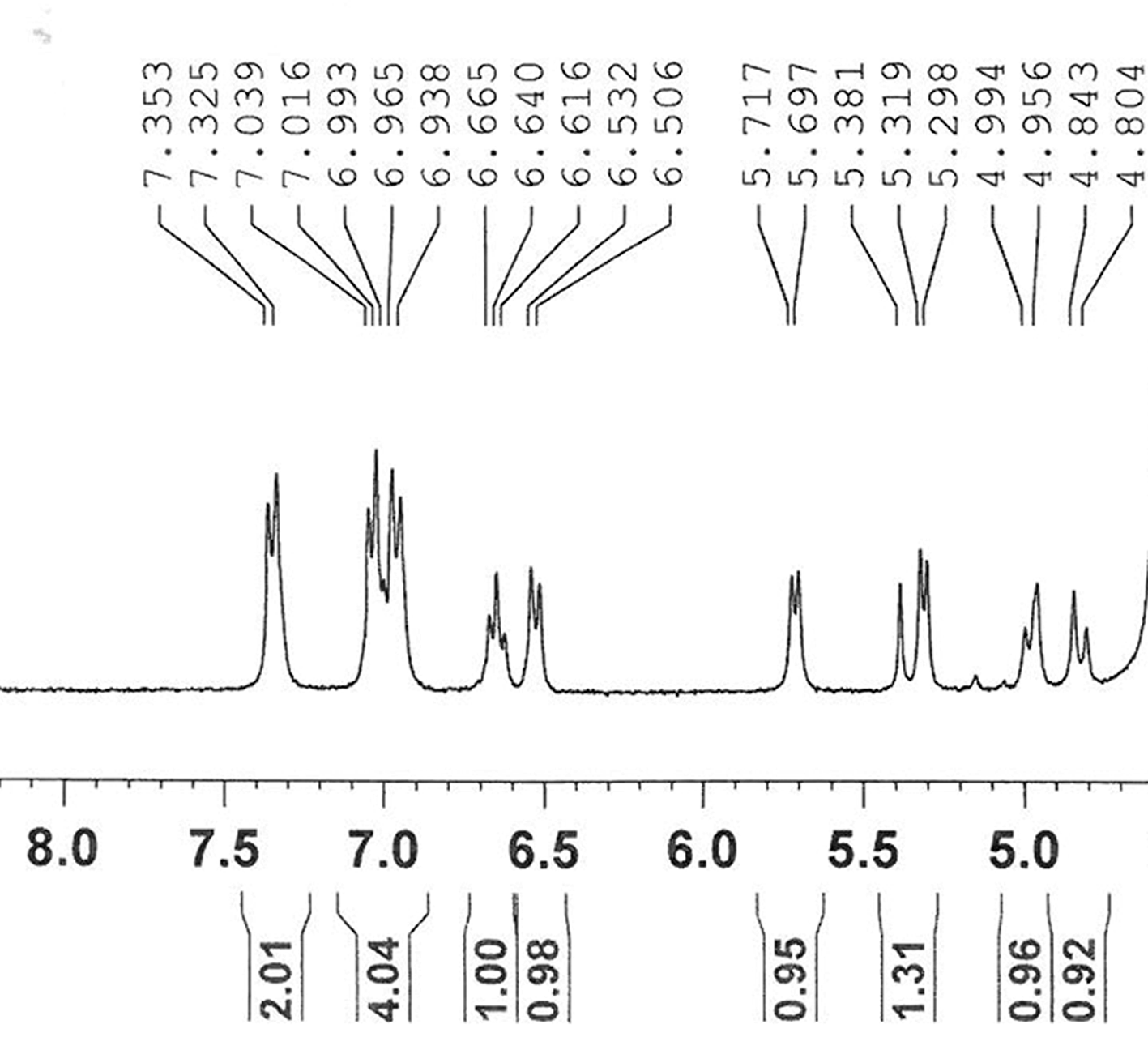


**Supplementary Scheme 2.** Partial ^1^H NMR spectrum of probe HCy-NBD in the presence of 25 equiv. of Na_2_SO_3_ in D_2_O : DMSO-d6 = 6 : 4.

**Supplementary Table 1.** Optical comparisons of probe Hcy-NBD with Hcy-D.

| Probe | Analyte(s) | System | Enhancement of I_green_/I_red_ | Space between red-green bands (nm) |
| --- | --- | --- | --- | --- |
| Hcy-D | HSO_3_^-^/SO_3_^2-^ | H_2_O-DMF (7-3) | 23-fold | 52 |
| HCy-NBD | HSO_3_^-^/SO_3_^2-^ | H_2_O-EtOH (6-4) | 61-fold | 60 |


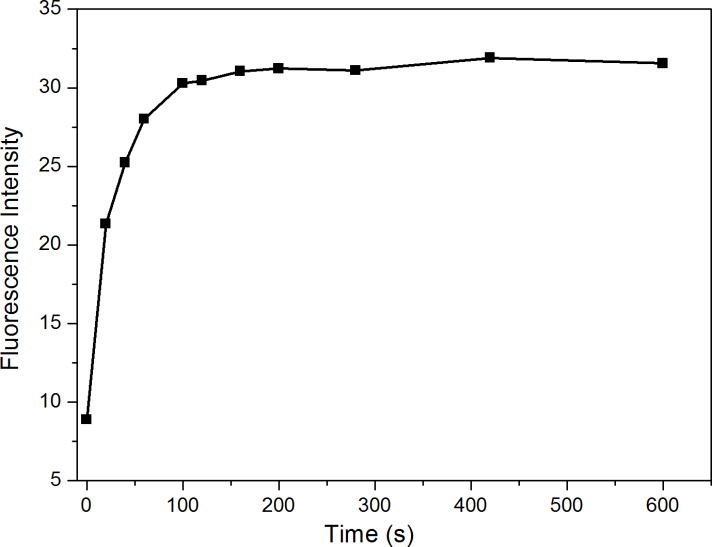


**Supplementary Figure 1.** Fluorescence intensity at 535 nm of HCy-NBD (10 μM) in the presence of NaHSO_3_ (250 μM) at different time intervals.


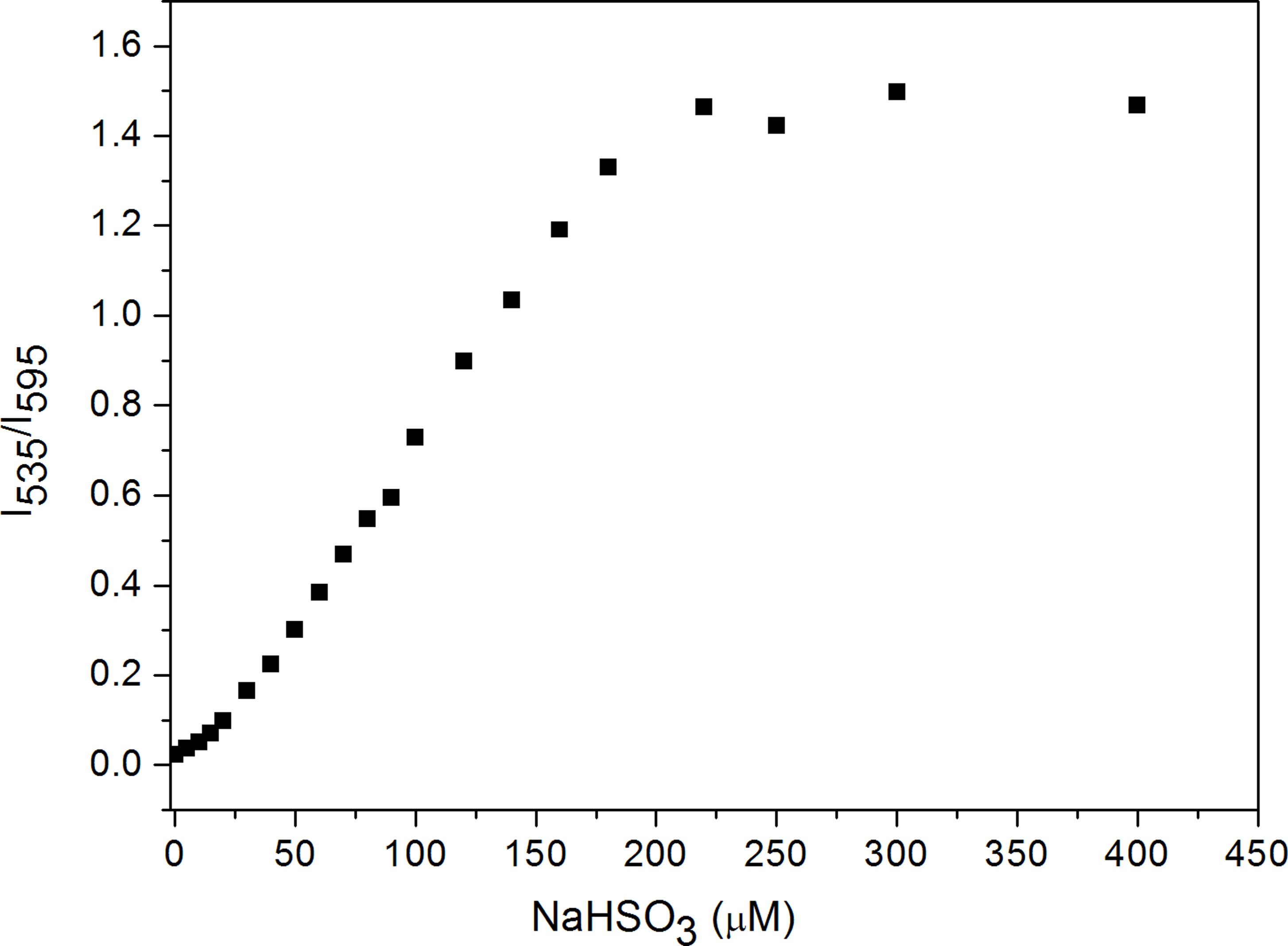


**Supplementary Figure 2.** Fluorescence ratio changes (I_535_/I_595_) of HCy-NBD (10 μM) in the presence of NaHSO_3_ (0-400 μM).


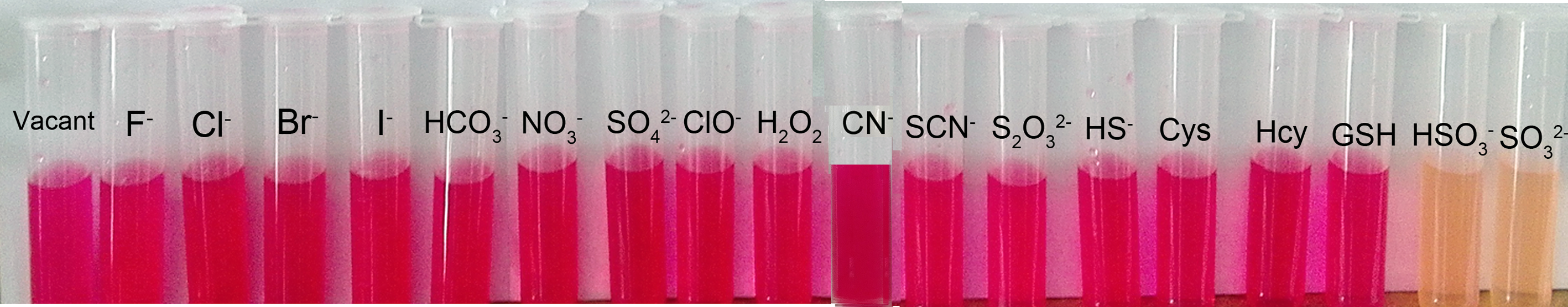


**Supplementary Figure 3.** HCy-NBD (10 μM) was treated with common anions (250 μM) and biothiols (1 mM) for 30 min. The photo was taken under room light.


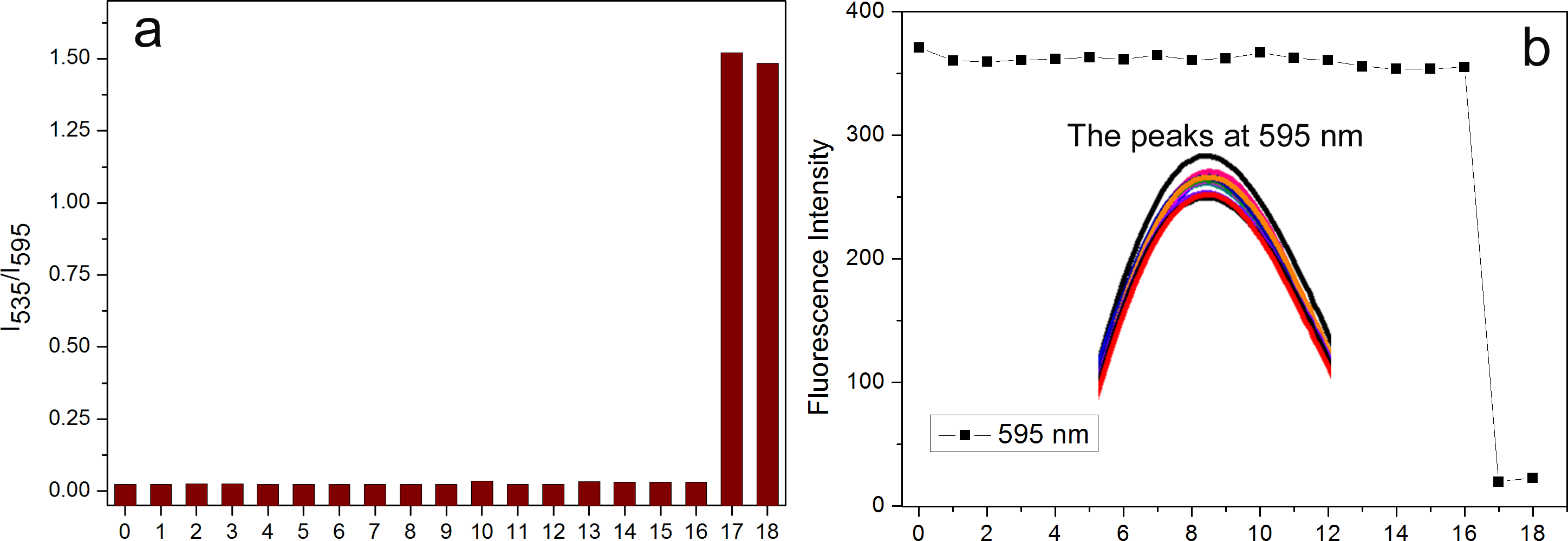


**Supplementary Figure 4.** Selectivity of probe HCy-NBD (10 µM) to various anions: 0. HCy-NBD alone; 1. F^-^; 2. Cl^-^; 3. Br^-^; 4. I^-^; 5. HCO_3_^-^; 6. NO_3_^-^; 7. SO_4_^2-^; 8. ClO^-^; 9. H_2_O_2_; 10. CN^-^; 11. SCN^-^; 12. S_2_O_3_^2-^; 13. HS^-^; 14. Cys; 15. Hcy; 16. GSH; 17. HSO_3_^-^; 18. SO_3_^2-^ (a) Fluorescence ratio (I_535_/I_595_) changes; (b) Fluorescence intensity at 595 nm. Inset: The expanded peaks at 595 nm in Fig. 4. Final concentration for all the species was 250 µM except for 14-16 (1 mM). ex = 345 nm, slit: 10 nm/12 nm.


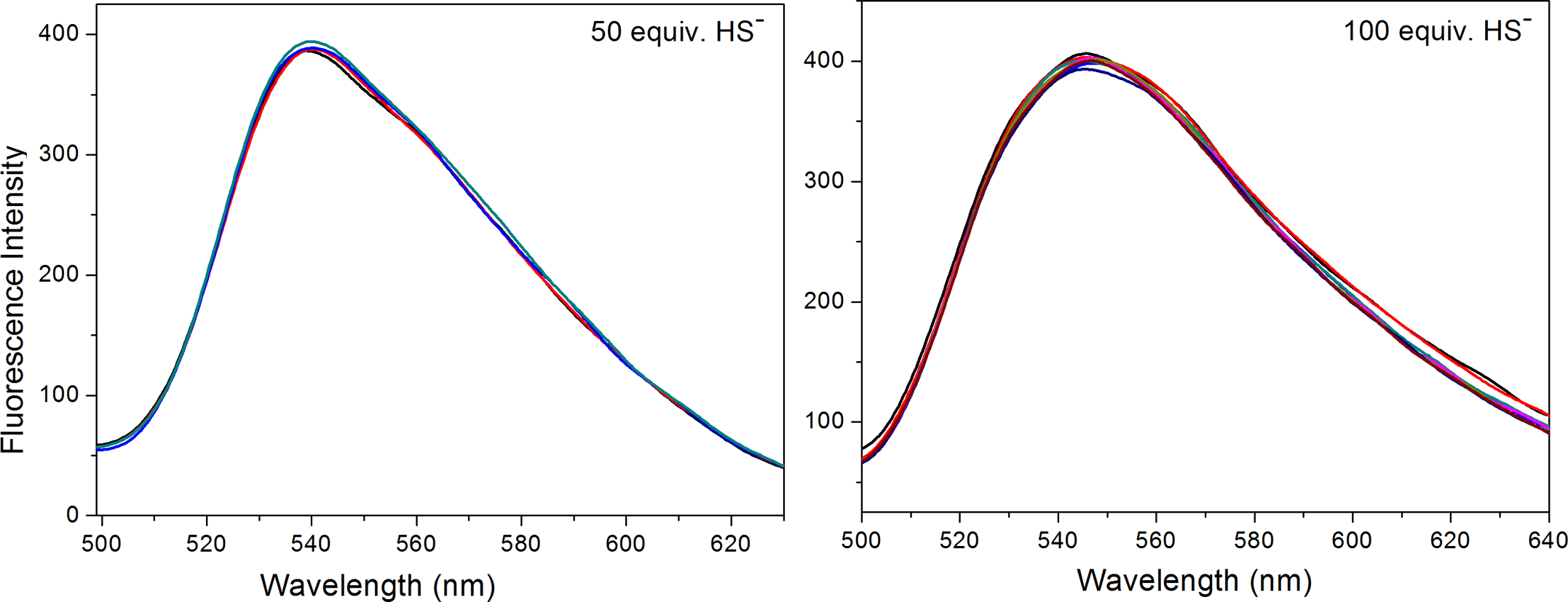


**Supplementary Figure 5.** Compound Donor (5 μM) was treated with HS^-^ (50 or 100 equiv.) in 1 h.


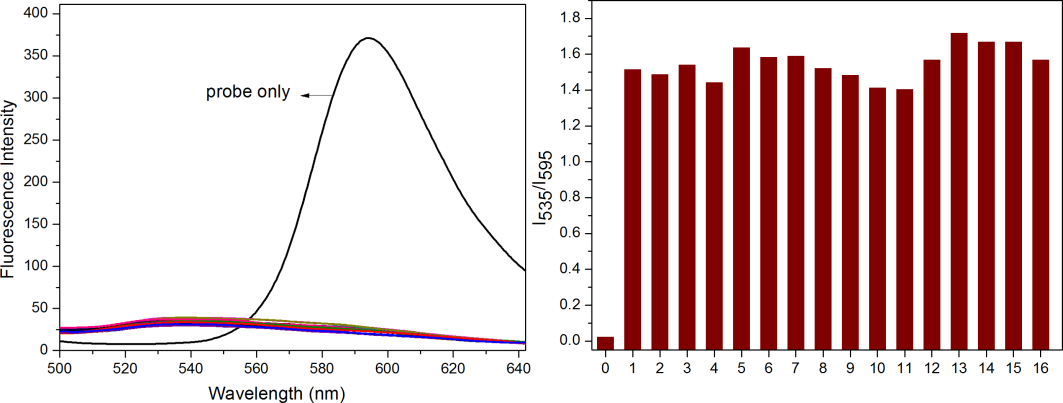


**Supplementary Figure 6.** Fluorescence response of HCy-NBD (10 µM) to NaHSO_3_ (250 µM) in the presence of various species: 0. HCy-NBD alone; 1. F^-^; 2. Cl^-^; 3. Br^-^; 4. I^-^; 5. HCO_3_^-^; 6. NO_3_^-^; 7. SO_4_^2-^; 8. ClO^-^; 9. H_2_O_2_; 10. SCN^-^; 11. S_2_O_3_^2-^; 12. HS^-^; 13. Cys; 14. Hcy; 15. GSH; 16. SO_3_^2-^. Final concentration for all the species was 250 µM except for 13-15 (1 mM). ex = 345 nm, slit: 10 nm/12 nm.


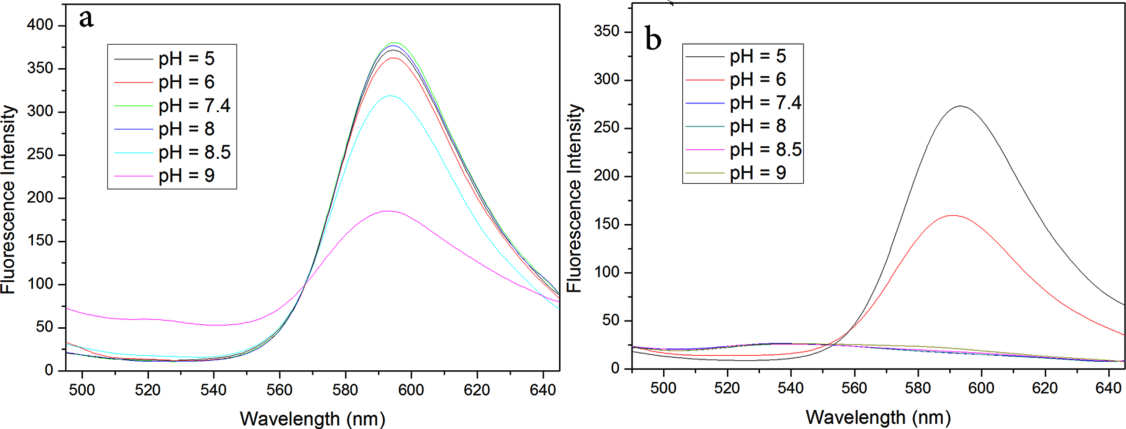


**Supplementary Figure 7.** (a) The effect of pH on the fluorescence of HCy-NBD (5 μM); (b) The fluorescence spectra of HCy-NBD (5 μM) in the presence of NaHSO_3_ (25 equiv.) in different pH solutions.


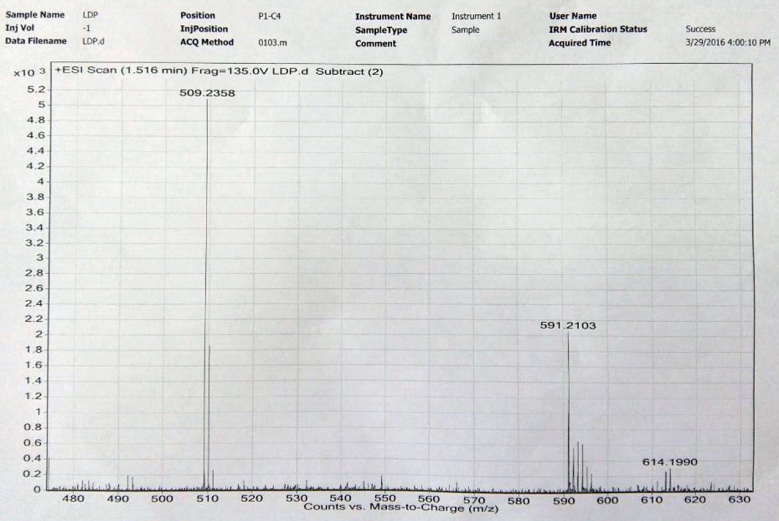


**Supplementary Figure 8.** High-resolution mass spectroscopy of HCy-NBD (10 μM) in the presence of NaHSO_3_ (250 μM).


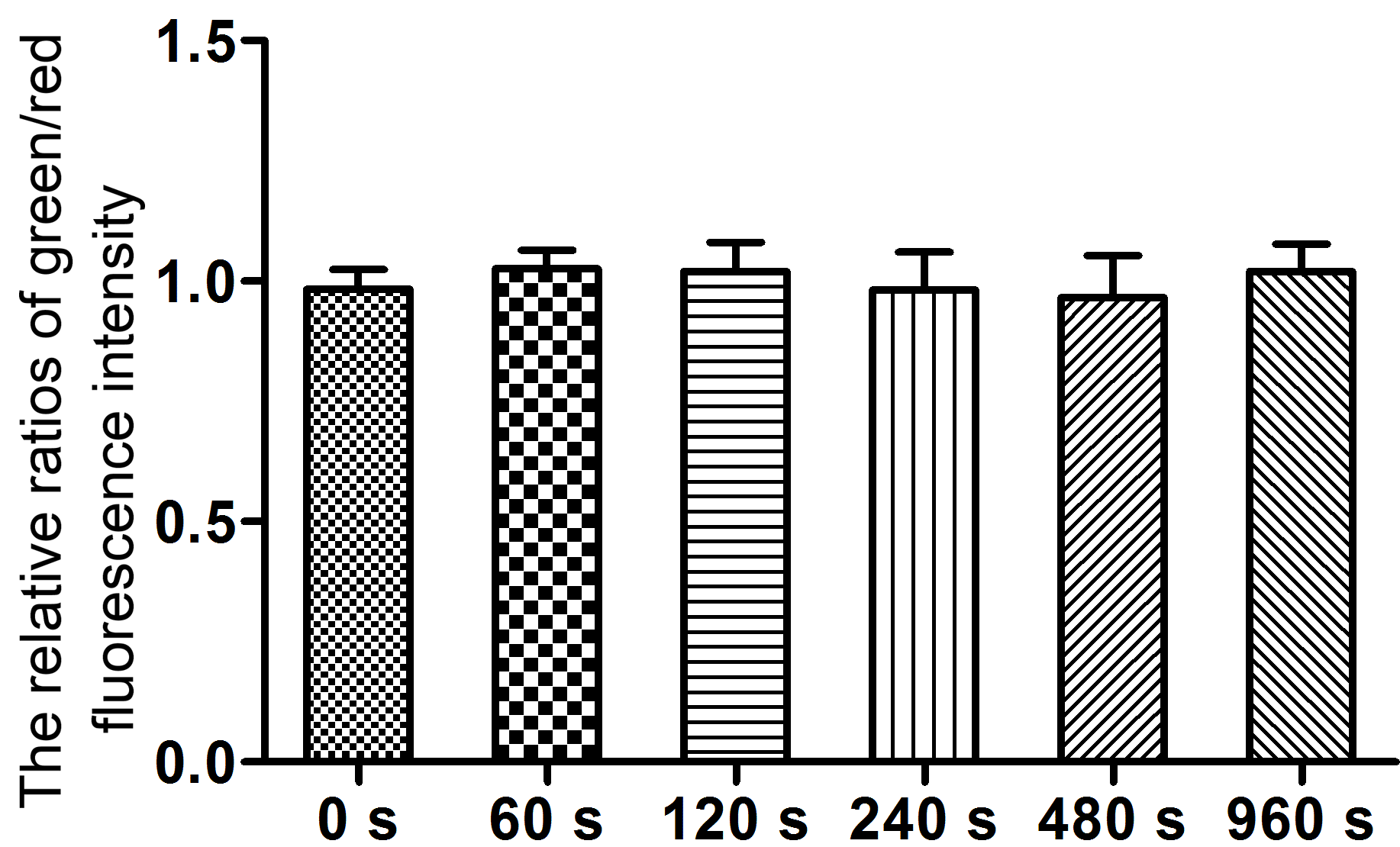


**Supplementary Figure 9.** Photostability of probe HCy-NBD in living Hela cells. The cells were incubated with HCy-NBD (5 µM) for 1 h beforehand.


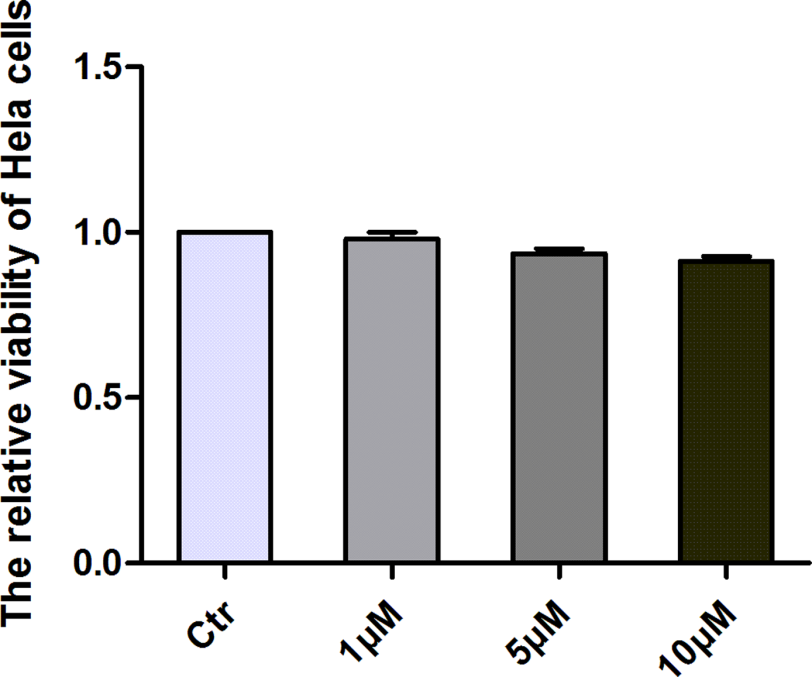


**Supplementary Figure 10.** Cell viability by a standard SRB assay. Hela cells were incubated with HCy-NBD (1, 5 or 10 µM) for 6 h, (n = 3).


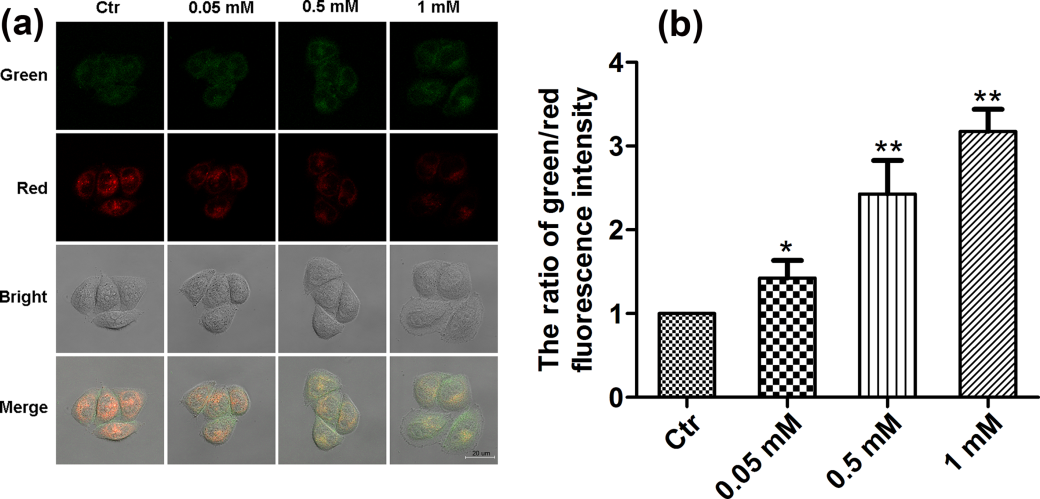


**Supplementary Figure 11.** (a) Fluorescence and bright field images of Hela cells incubated with HCy-NBD (5 µM) for 1 h, then with NaHSO_3_ (0, 0.05, 0.5, 1 mM) for another 0.5 h. (b) The relative ratios of green/red fluorescence intensity. Images were acquired from 405-555 nm for green fluorescence, and from 560-700 nm for red fluorescence, respectively, * p < 0.05%; ** p < 0.01%, n = 3.

**
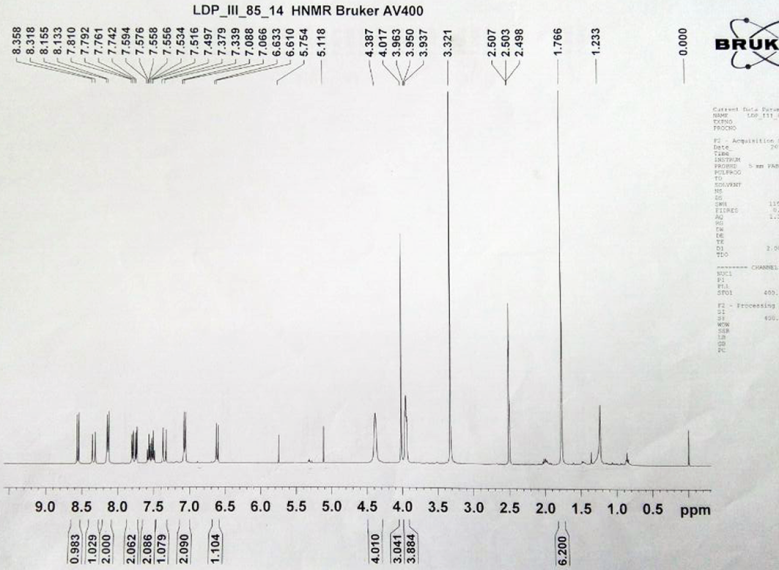
**

**Supplementary Figure 12.** ^1^H NMR spectrum of HCy-NBD in DMSO-*d*_6_.


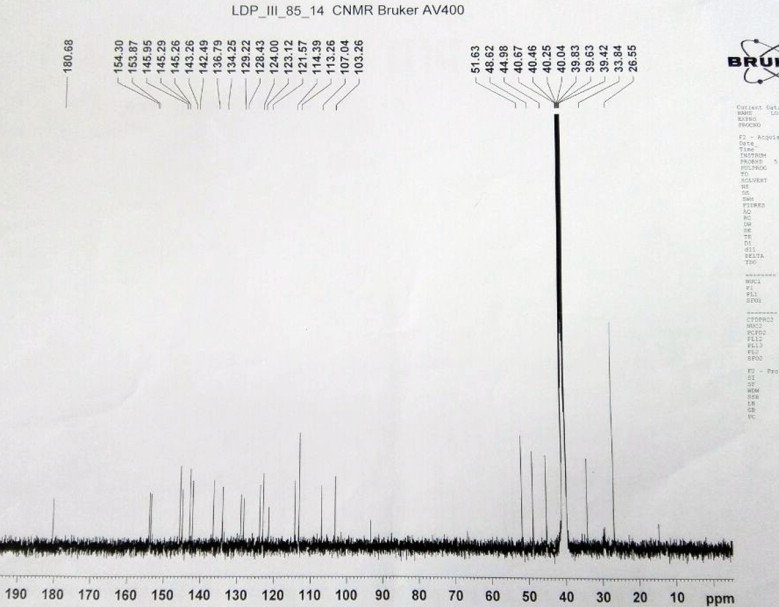


**Supplementary Figure 13.** ^13^C NMR spectrum of HCy-NBD in DMSO-*d*_6_.


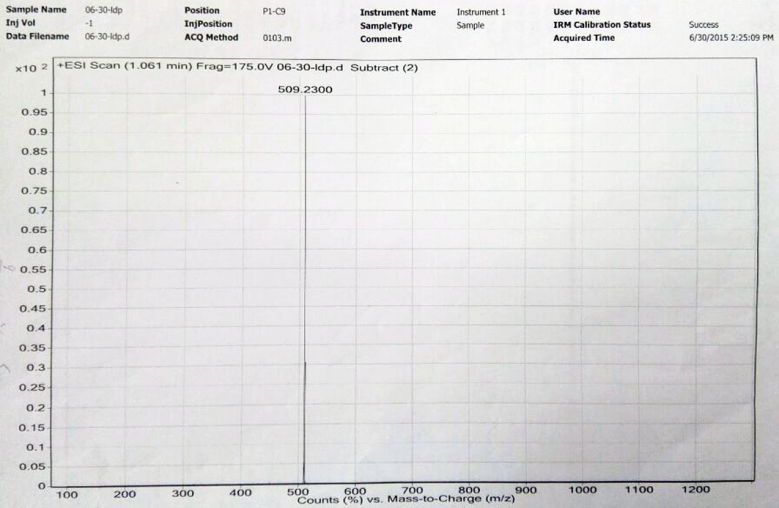


**Supplementary Figure 14.** HRMS spectrum of probe HCy-NBD.


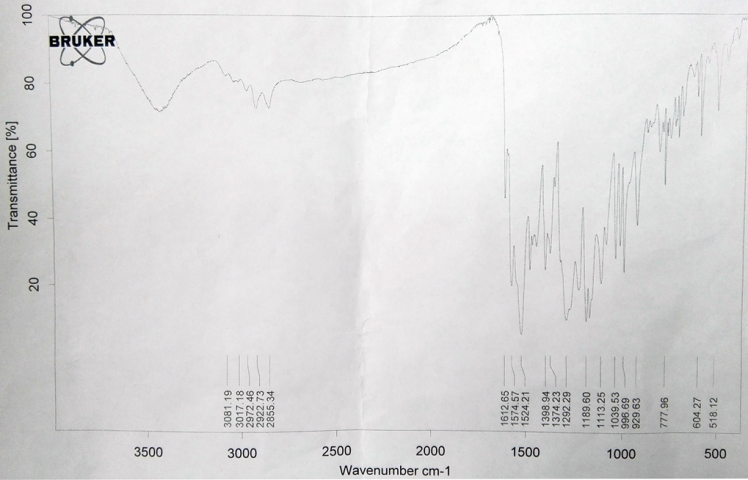


**Supplementary Figure 15.** FT-IR spectrum of probe HCy-NBD.


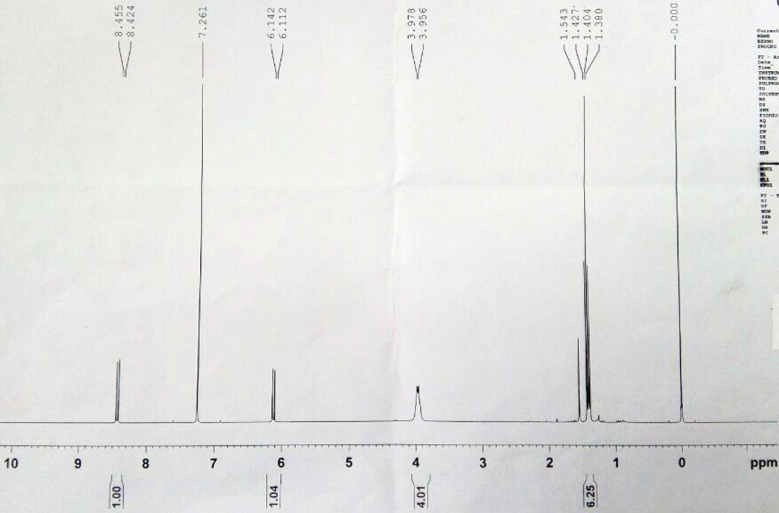


**Supplementary Figure 16.** ^1^H NMR spectrum of the **Donor** in CDCl_3_.


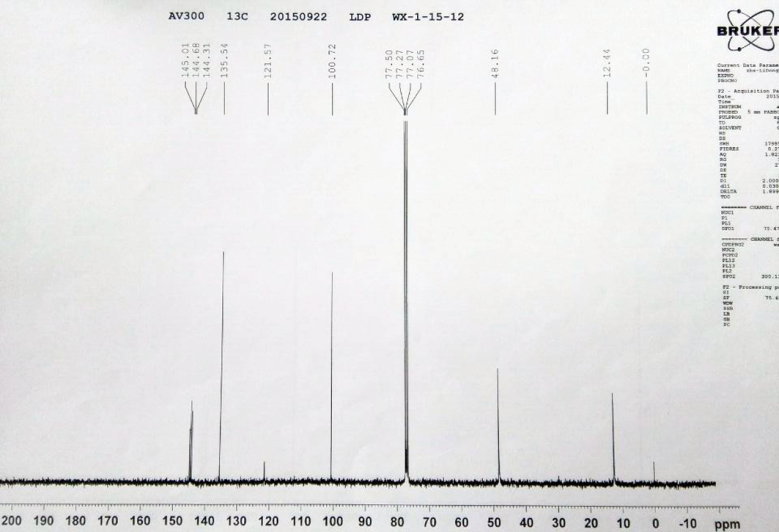


**Supplementary Figure 17.** ^13^C NMR spectrum of the **Donor** in CDCl_3_.


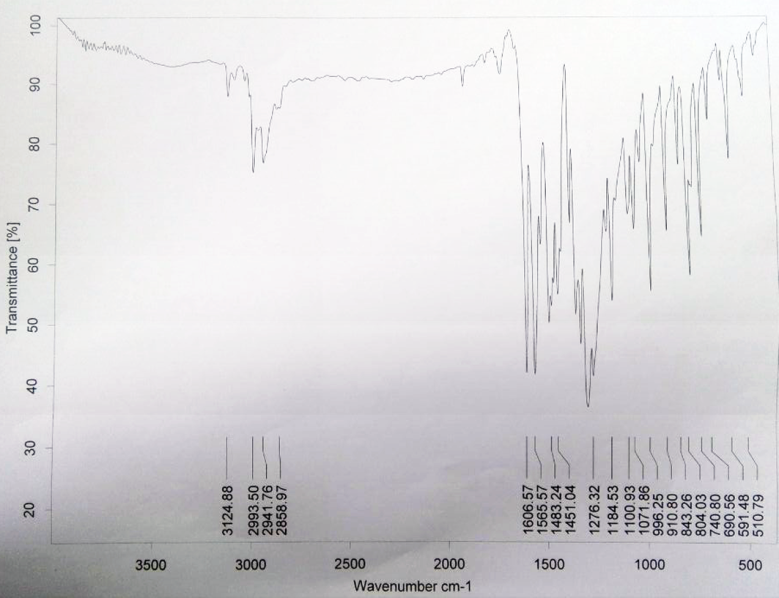


**Supplementary Figure 18.** FT-IR spectrum of the **Donor.**
